# Measuring the effects of pedagogical agent cognitive and affective feedback on students’ academic performance

**DOI:** 10.3389/frai.2024.1495342

**Published:** 2024-12-16

**Authors:** Marta Arguedas, Thanasis Daradoumis, Santi Caballé

**Affiliations:** ^1^IN3-Department of Computer Science, Multimedia and Telecommunications, Open University of Catalonia, Barcelona, Spain; ^2^Escuelas Universitarias Gimbernat (EUG), adscrita a la Universitat Autònoma de Barcelona, Sant Cugat, Spain; ^3^Department of Cultural Technology and Communication, University of Aegean, University Hill, Mytilini, Greece

**Keywords:** intelligent tutoring systems, affective tutor, pedagogical agent, cognitive feedback, affective feedback

## Abstract

There is still a debate on the influence and effectiveness of pedagogical agents in a learning environment, especially on the means these agents employ for enhancing students’ academic performance. The current study aims at measuring the effectiveness of cognitive and affective feedback (CaAF) types that a human teacher and a virtual Affective Pedagogical Tutor (APT) used in their groups of students (control and experimental groups respectively) in an authentic long-term learning situation. Participants were a sample of 115 students carrying out collaborative activities in a “web design” course. Our findings showed that APT cognitive feedback (*CF*) significantly increased students’ learning outcomes compared to the human teacher’s feedback, whereas APT affective feedback (AF) only achieved partial success. Nevertheless, the study has some limitations: it is based on a single course and a specific academic context, limiting the generalizability of its findings. Additionally, while cognitive feedback demonstrated a clear impact, the analysis of affective feedback was less conclusive, and its design requires further refinement. Finally, the cross-sectional design of the study restricts the ability to assess whether improvements in learning outcomes persist over time. Future research directions include exploring the generalizability of results across diverse disciplines, deepening the analysis of affective feedback, and incorporating longitudinal studies to evaluate the durability of the observed effects.

## 1 Introduction

Emotions are critical for motivation, self-regulated learning, and performance, playing a vital role in cognitive development (Arguedas and Daradoumis, 2021). Research in affective learning focuses on emotion awareness, affective feedback, and emotional education to enhance learners’ skills in identifying, managing, and understanding emotions—both their own and others’. Effective emotional education promotes self-motivation, conflict recovery, and social–emotional connection to studies, peers, and instructors (D’Mello et al., 2011; Pérez-Marín, 2021). Tools that provide group awareness and facilitate collaboration are also key, aiming to empower students to complete their learning journey successfully (Devis-Rozental et al., 2017).

## 2 Literature review on affective PAs, cognitive and affective feedback

Α pedagogical agent (PA) is designed to guide learners through an educational environment with the aim at creating an interesting, pleasant, safe and creative environment for learning, but also assists learners to cope with learning difficulties, accomplish their learning objectives as well as enhance their self-reflection about what they learned and how they learned during the learning process (Arguedas and Daradoumis, 2021; Norman, 2004) and it caused them important changes in learning and motivation.

According to Kim et al. (2017), Multiple Intelligent Pedagogical Agents (MIPAs) are a group of intelligent agents integrated into an educational system designed to collaboratively interact with learners to support their learning processes. Each agent in the system typically has distinct roles, expertise, or characteristics that contribute uniquely to the instructional objectives. The agents can embody various personas, such as tutor, motivator, peer, or facilitator, providing diverse perspectives and fostering a rich and engaging learning environment. Some approaches used MIPAs to promote more flexible and dynamic affective communication. These systems were designed to adapt to the cognitive and affective needs of the learner (Ammar et al., 2010). They also aimed to detect and process users’ emotions, enabling real-time responses to user needs. This allowed the systems to provide more complex motivational feedback (Scholten et al., 2017). Such feedback was perceived positively by students because it supported their learning and motivation (Kim et al., 2017). More recent reviews have found that the presence of PAs can improve learning outcomes. However, the effectiveness of different feature combinations and outcome variables has not been systematically studied. As a result, it remains unclear which features work best or under what circumstances (Martha and Santoso, 2019; Arguedas and Daradoumis, 2021).

To better understand how students perceive the quality of the cognitive feedback (*CF*) they receive, several researchers highlighted the importance of the content of feedback (Kornell and Rhodes, 2013; Van der Kleij et al., 2015): global versus elaborated. Global feedback may allow students to verify the correctness of their answers or indicate whether the answer is correct or incorrect. In contrast, elaborated feedback (e.g., providing additional information, extra study material, or an explanation, giving a hint or an example) can offer, detailed and constructive information that engages students into more effective cognitive processes, which enables learners to perform better in subsequent tasks (Finn et al., 2018; Wang et al., 2019). *CF* in our PA uses elaborated feedback of different types which are presented in detail in Section 4.1. The effectiveness of *CF* depends on task difficulty, learners’ characteristics (e.g., age, prior knowledge), feedback type and format (Attali et al., 2016; Lin et al., 2020). Therefore, more work is needed to explore students’ perception of *CF* quality and how it affects learning development in a computer-based environment.

Early e-learning systems began integrating affective feedback to improve learner motivation and mood (Mao and Li, 2009). Different strategies have been used, such as empathetic responses or task-based adjustments, to align with learners’ emotions (Robison et al., 2009). D’Mello et al. (2011) introduced AutoTutor, an agent that adapts feedback based on learners’ cognitive and emotional states, promoting engagement. Bevacqua et al. (2012) describe systems that select and analyzes feedback based on verbal (like tone of voice and word choice) and non-verbal (such as facial expressions, gestures, and posture) cues by leveraging emotion recognition techniques and embodied conversational agents. For instance, if a learner shows signs of frustration (e.g., frowning, slumped posture, or using negative language), the system might adapt its response by offering empathetic and supportive feedback. By dynamically interpreting these cues, the system aims to adjust its interactions in real-time, providing feedback that aligns with the user’s emotional state, thereby fostering a more engaging and supportive learning environment.

Studies show that affective feedback can boost motivation and enjoyment but depends on the believability of the agent (Guo and Goh, 2016). Existing studies, however, mainly focus on motivation and satisfaction, lacking a comprehensive exploration of other emotional responses (Lin et al., 2020).

Other emotional responses are critical in shaping effective learning experiences. On the one hand, frustration can hinder persistence and problem-solving, but personalized feedback strategies help mitigate its effects and improve engagement (Rajendran et al., 2019). Likewise, anxiety negatively impacts cognitive performance; yet embodied agents can reduce anxiety and foster a supportive learning environment (Kim et al., 2017). On the other hand, empathy plays a vital role in enhancing collaborative learning by promoting emotional connections among peers (D'Mello and Graesser, 2012). And resilience enables learners to view mistakes as growth opportunities, fostering a mindset focused on continuous improvement (Yeager and Dweck, 2012). These emotions highlight the importance of tailored affective feedback in education. Research suggests that emotion regulation strategies, such as reappraisal, can help learners manage negative emotions, increasing engagement (Malekzadeh et al., 2015).

Text-based feedback remains popular due to its accessibility, and its effectiveness is influenced by clarity and timely delivery (Howard, 2021). Affective support helps reduce off-task behavior and boredom, contributing to improved learning outcomes (Grawemeyer et al., 2017). For instance, systems can provide motivational prompts or empathetic messages when disengagement is detected, helping students refocus and stay productive. Gamification elements also enhance engagement through personalized feedback, particularly when addressing frustration (Rajendran et al., 2019). Features like rewards and adaptive challenges turn frustration into motivation, promoting persistence and a sense of achievement. Based on the review of the literature we adapted various feedback types to the context of our study, the age of participants and learning situation (group activity on “web design” conducted in the class laboratory). The resulting list of CaAF types is presented in Table 1, Section 3.

**Table 1:** The cognitive and affective feedback types provided in the teaching sessions.

Cognitive feedback		Affective feedback	
Make the course objectives clearer and more understandable “Be aware that one of the course objectives is to design a consistent website. By designing with consistency you’ll learn how to create interfaces which build trust and teach users repeatable patterns that help them work through your site much quicker.”	3.1	Create a satisfactory working climate in the group “Trust the abilities of your peers and encourage conversations on any problem they have.” HT: “Look at this short video and think how this group of students interacts and feels working together.”	3.9
Provide students appropriate and complementary information to increase their ability to complete their work “Hypertext Markup Language (HTML) is the standard markup language for documents designed to be displayed in a web browser. Please look at the window; there you will find all the information you are looking for. If you still need anything else, please let me know.”	3.2	Be subtle enough not to interfere and affect the duration of the course negatively “Keep on contributing smoothly and to the point so that you do not distract your groupmates from completing your common goals. You need to complete your project on time.”	3.11
Organize and present the contents in a more orderly manner “Please plan specific topics under a category and get pretty granular with what you’d like to include.” HT: “In this case, you better provide your findings in the form of a table rather than as a plain text.”	3.3	Guide students to better communicate their individual results in the group “There is a lack of effective communication in your group. You need to communicate your individual results in the group as soon as you have them. This will strengthen confidence between each other and reduce anxiety.”	3.12
Build on students’ existing knowledge based on their level and needs “So far you have created some good menus. For larger navigation menus you might have to add sub-menus or links in a larger list.”	3.4	Help students complete the activity successfully “Do not worry about making mistakes. You can always learn from them and they can ultimately lead you to achieve a better design.” HT: “Be more critical with your choices and sincere with the choices of your teammates. This will ultimately enhance your confidence for achieving a better Web page design.”	3.13
Enrich the knowledge presented with novel elements “Keep in mind that users should understand a lot about your site just from the header. This area should explain what the site does and what it’s about, not to mention the top navigation links.” HT: “If you write original content, search engines will help your site get more exposure.”	3.5	Support students to deal with the final evaluation successfully “Never let the fear or uncertainty prevent you from facing the last step in this process. The excellent work you have performed guarantees the best in the final assessment.”	3.15
Enable students to work more effectively in small groups “In order to design a well-structured website you need to set and share clear objectives.”	3.6	Help students acquire skills and attitudes “Make a list of what you have learned and find the good points of view you have developed during this work so far. Remember that these little actions can add up into big change.”	3.16
Foster individualized learning within working groups “Before passing to the next stage of your design, each of you should reflect about what you learned (what has changed with respect to your initial ideas and knowledge), and how you learned (what led you to change your points of view).”	3.7	Enable students to better face their difficulties “Learning comes from facing your difficulties. Dare to try a different option. Ask help from your peers. Look at a best practice more thoroughly.” HT: “Keep on confident and persistent with the principle of simplicity. You’re doing a pretty good job so far!”	3.17
Provide more support to practical aspects “Do not you think that your webpage has ‘flashy’ objects that get in the way of browsing? Beware of unnecessary elements such as pop-ups.” HT: “Wouldn’t it be cool to have the logo on the right side of the screen?”	3.8	Offer students possibilities to make the best decision in cases of doubt “Do not ignore your doubts but neither stay stuck in them. Elaborate on the best options and choose the most appropriate one to go on. Ask your peers’ opinion as well.”	3.18
Support students’ learning with effective instructional procedures “At this point, you should form pairs to discuss your results and findings and then share your conclusions within your group.”	3.10	Trigger and maintain students’ interest in the activity and their learning “Look at this website. Do not you find its design curious”? Then, look at this other design. Does it provoke you to change as many things as you can?”	3.19
Ensure the accomplishment of the learning objectives according to the criteria set by the course “Make sure that you design a consistent website. The consistent style and multi-link menus are great for big sites and blogs. As users get familiar with those links they’ll have an easier time browsing through content.” HT: “You should provide more specific principles of creating an effective web page, including an in-depth consideration of information architecture.”	3.14		

## 3 Research aims

### 3.1 Aim

The aim of this study has been to examine whether the APT CaAF increased significantly the students’ learning outcomes compared to human teacher’s feedback.

In order to achieve the above goal, we performed an experiment that we included in an existing class in a classroom setting. In particular, together with the class teacher we designed a scenario which involved an authentic learning experience through problem-based learning coupled with collaborative learning.

In this context, the APT is a specific agent whose design has followed the Activity Theory Framework (Engeström et al., 1999), and forms part of a larger project and framework which includes several components. This framework involves an emotion analysis model which first analyzes text and conversation (wiki, chats and forum debates) generated by students involved in collaborative learning activities. Then, it proceeds to identify and represent the students’ emotions that take place during these activities in a non-intrusive way. This information is shown to both the human teacher and the APT, thus providing *emotion awareness* with regard to the way students’ emotions appear and evolve over time. This enables both the teacher and the APT to offer students cognitive and affective feedback that influences students’ motivation, engagement, self-regulation and learning outcome. Details of how the APT and feedback work are fully described in the aforementioned research articles (Arguedas and Daradoumis, 2021).

Since the distinction between CaAF is central to our research, we would like to make clear how APT treats each feedback type. As students work in the Moodle environment, they may raise cognitive doubts about the topic or the activity to be carried out. Then the APT responds through *CF* using spoken and/or written language which provides the student the necessary information about the question at hand.

If the student questions do not correspond to the topic or the activity they are carrying out, or if they have impolite, inappropriate, or distracting tone, the APT responds by giving AF with the aim to redirect student’s behavior, as well as their attention to the activity they must carry out. We set the following research questions for the specific learning situation.

### 3.2 Research questions

The main research questions we deal in this work are the following:

1. Has the APT *CF* increased significantly students’ learning outcomes compared to human teacher’s feedback?
2. Has the APT AF increased significantly students’ learning outcomes compared to human teacher’s feedback?

### 3.3 Definition of variables for the learning situation

The learning situation represents the space where the main teaching and learning processes occur. There are two independent variables (IVs) relevant to the study: Affective Feedback (A) and Cognitive Feedback (C). As such, the weight of each variable is the same, that is, both variables A and C are equally important for the learning situation at hand. The IVs include the types of cognitive and affective feedback (CaAF) provided by the APT (Affective Pedagogical Tutor) and the human teacher. These variables are qualitative but are indirectly measured through their implementation in the experimental design (e.g., elaborated feedback, motivational feedback).

The dependent variables (DVs) are the students’ learning outcomes, which are measured using a 5-point Likert-type scale, ranging from 1 (Almost never) to 5 (Almost always). The scale captures students’ perceptions of the effectiveness of the feedback they received, including its impact on their learning experience.

The study uses a questionnaire to quantify the dependent variable (learning outcomes) by associating specific types of CaAF with student feedback ratings. Statistical analyses, including t-tests, are applied to compare the means of these responses across the control and experimental groups.

## 4 Method

### 4.1 Participants and procedure

Participants were a sample of 115 students attending the course “Web Design.” We randomly divided students in two big groups, a control and an experimental group, with 57 and 58 students, respectively. Then, each big group was further divided into smaller teams. We wanted the teams to be big enough so that to promote more interaction in the team forums. For this reason, we created teams between 4 and 7 members. More specifically, in the control group (supervised by the human teacher), 12 teams were formed randomly: nine teams of four members and three teams of seven members. In the experimental group (where the APT was acting), 16 teams were also formed randomly: three teams of seven members, one team of five members and eight teams of four members. In this sense, the groups have been randomly distributed; both samples have been independent and had a normal distribution.

**Figure 1:**
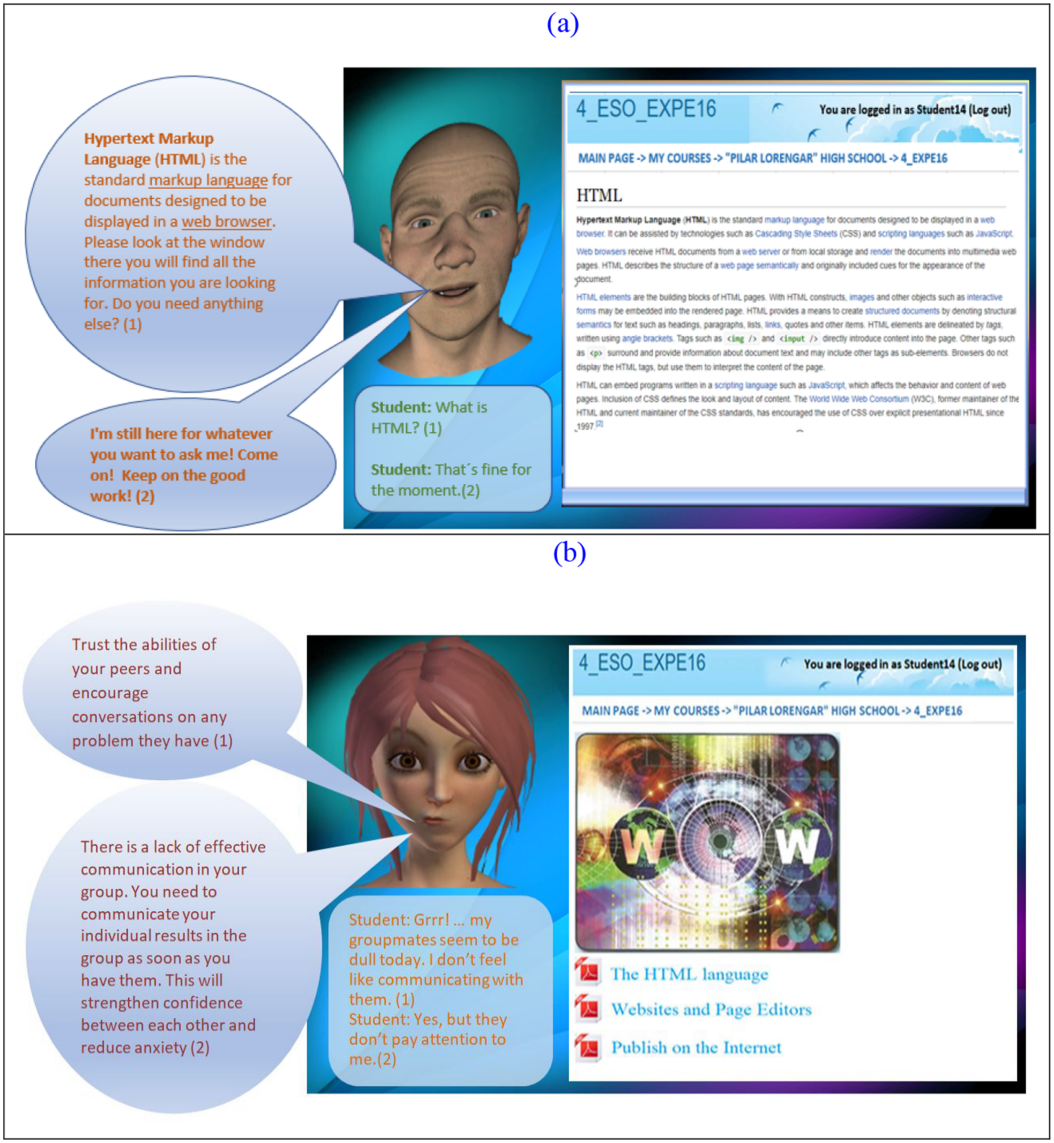
Example of APT cognitive feedback **(A)** and APT affective feedback **(B)**.

As mentioned in Section 2, our APT is a specifically designed agent that is based on a work project and framework. This framework involves a cognition-emotion analysis model which is composed of two tools. One of them, the fuzzy logic tool, analyses students’ text to infer a dimensional and categorical emotional state of the students during their learning process. The other one, the APT, is a client–server web application. A client is installed in each student’s computer that connects to the server. When running on student’s computer, it displays an environment with the APT on the left side of the screen, an embedded Moodle LMS on the right side of the screen, as well as a text edit box at the bottom that allows the student to contact the APT textually. As such, the APT is characterized by a specific voice, the emotional expressions that can display and the dialogs that can be involved. The student works on the LMS, carries out his/her tasks, collaborates with peers, whereas at the same time he/she can interact with the APT in a textual manner through the edit box located at the bottom of the screen. The APT responds to the student with audible and gestural signals that were scheduled in advance, while providing the student with the information that he/she previously requested (Arguedas and Daradoumis, 2021). Two examples of this responds are shown in Figure 1.

Students’ emotional states were detected by our emotion analysis model after each student intervention (message) in the group forum. This information was used to define both the teacher and the APT reaction to each student, giving them CaAF. The types of CaAF provided are described in Table 1. They represent generic types of feedback. Since both the human tutor and APT act independently, each provides its own particular feedback in its own wording and expression, i.e., feedback articulation differs between the control and experimental groups; however, each particular feedback utterance should adhere to the generic feedback type it refers. It means that every instance of feedback given during the study was designed to fit within one of the predefined categories of cognitive or affective feedback. This consistency ensures that both the human teacher and the Affective Pedagogical Tutor (APT) adhered to the same framework for feedback delivery, maintaining the reliability of comparisons between the experimental and control groups. This structured alignment was central to evaluating the distinct impacts of cognitive and affective feedback types on student learning outcomes. This is a condition that the human teacher was aware of. As such, the kind of support the teacher was giving to students had to be associated with a specific feedback type. For the sake of illustration, in Table 1 we show examples of all CaAF types provided by the APT in our learning situation.

### 4.2 Data collection

The questionnaire was composed of questions that were associated with the 19 CaAF types (one question for each feedback type) presented in Table 1. For all questions, we used a five-point Likert-type scale ranging from 1 (Almost never) to 5 (Almost always) requiring a quantitative answer. The aim of the questionnaire was to measure the dependent variable, ‘students’ learning outcomes.’ To do so, we look how successful the human teacher’s and APT CaAF have been. The comparison of the mean values of this feedback can provide this information; this is completed by a Student’s t-test as well [as it is shown in section 5.1 -Table 2 (a) and section 5.2 -Table 2(b)].

**Table 2:** Mean values of students’ learning outcomes and T-test related to (a) cognitive and (b) affective feedback.

(a) Cognitive feedback										
	Control Group (*N* = 57)				Experimental Group (*N* = 58)				Levene’s test for equality of variances (1)	t-test for equality of means (2)
	Mean (SD)	Min–Max	Median	Mode	Mean (SD)	Min–Max	Median	Mode		
3.1	3.58 (1.387)	1–5	4	5	4.27 (0.55)	3–5	4	4	0.000 (b)	0.052
3.2	3.16 (1.068)	1–5	3	3	4.05 (0.785)	2–5	4	4	0.137 (a)	0.004
3.3	3.26 (1.368)	1–5	3	3	4.18 (0.588)	3–5	4	4	0.001 (b)	0.012
3.4	3.21 (1.228)	1–5	3	3	4.23 (0.612)	3–5	4	4	0.006 (b)	0.003
3.5	3.16 (1.463)	1–5	3	4	4.23 (0.612)	3–5	4	4	0.000 (b)	0.007
3.6	3.63 (1.212)	1–5	4	3	4.18 (0.664)	3–5	4	4	0.003 (b)	0.089(*)
3.7	3.05 (1.353)	1–5	3	4	3.77 (0.752)	2–5	4	4	0.007 (b)	0.049
3.8	3.16 (1.119)	1–4	4	4	4.18 (0.501)	3–5	4	4	0.002 (b)	0.001
3.10	3,37 (1.165)	1–5	3	3	4.09 (0.811)	2–5	4	4	0.050 (b)	0.030
3.14	3.58 (1.121)	1–5	4	4	4.23 (0.528)	3–5	4	4	0.002 (b)	0.030

(b) Affective feedback										
	Control Group (*N* = 57)				Experimental Group (*N* = 58)				Levene’s test for equality of variances (1)	t-test for equality of means (2)
	Mean (SD)	Min–Max	Median	Mode	Mean (SD)	Min–Max	Median	Mode		
3.9	3.53 (1.219)	1–5	4	4	4.14 (0.71)	2–5	4	4	0.013 (b)	0.065(*)
3.11	3.37 (1.383)	1–5	4	4	4.09 (0.75)	2–5	4	4	0.002 (b)	0.052(*)
3.12	3.11 (1 15)	1–5	3	3	4.14 (0.64)	2–5	4	4	0.021 (b)	0.002
3.13	3.47 (1 219)	1–5	4	4	4.18 (0.733)	2–5	4	4	0.018 (b)	0.035
3.15	3.53 (1 389)	1–5	4	4	4.00 (0.69)	2–5	4	4	0.001 (b)	0.189(*)
3.16	3.53 (1 264)	1–5	4	3	4.05 (0.785)	2–5	4	4	0.015 (b)	0.132(*)
3.17	3.16 (1 015)	1–4	3	4	4.05 (0.844)	2–5	4	4	0.167 (a)	0.004
3.18	3.26 (1 485)	1–5	4	4	3.95 (0.722)	2–5	4	4	0.000 (b)	0.076(*)
3.19	3.26 (1 447)	1–5	4	4	4.05 (0.722)	3–5	4	4	0.001 (b)	0.042

(1) Results obtained from Levene’s test; a, Equal variances are assumed; b, Equal variances are not assumed.

(2) Results obtained from t-test.

(*) There are not significant differences between CG and EG.

In addition, students’ academic achievement, which is a qualitative outcome obtained from different evaluation techniques such as observation or oral examinations, has been also consulted. For all questions, we used a five-point Likert-type scale ranging from 1 (Almost never) to 5 (Almost always) requiring a quantitative answer.

### 4.3 Research analyses

Apart from descriptive statistic measures, differences in student’s learning outcomes were examined through t-test for independent groups according to CaAF provided by the human teacher (control group) and the APT (experimental group).

Due to space restrictions, we provide a compact version of reliability statistics and multivariate normality measures instead of presenting them for each subscale. To ensure the reliability of data collection, the Cronbach’s alpha coefficient has been applied to both groups, CG and EG as mentioned before, by obtaining values higher than 0.70, which reinforces the reliability of our indicators.

As the variables under study are quantitative, specifically, on a Likert scale of 1 to 5, the Student’s t-test for independent samples has been applied to analyze whether there are differences in the results obtained between the control and experimental groups, in the variables involved in the study: CaAF. To this end, the necessary hypotheses (normality of the data and homogeneity of variances) were previously verified. The confidence level chosen for the different tests is 95%. The Kolmogorov–Smirnov (KS) test was also used to test the normality of the different variables in each group. KS was not significant therefore normality was met. In addition, the skewness and kurtosis of each variable were examined to check for multivariate normality. Critical values of all test statistics were calculated. The results showed that data were normally distributed as absolute values of skewness and kurtosis did not exceed the allowed maximum (2.0 for univariate skewness and 7.0 for univariate kurtosis). The application of the Levene test for equality of variance defined the outcomes to be considered. Levene’s test for equality of variances tells us whether we can assume equal variances or not, i.e., if the probability associated with the Levene statistic is >0.05 we assume equal variances and if <0.05 we assume different variances. To that end, we establish the null hypothesis, Ho: “The APT feedback did not significantly enhance students’ learning outcomes compared to human teacher’s feedback.” Then, based on the t-test for independent groups, if Sig. (*p*-value) ≤ 0.05, Ho is rejected.

## 5 Results

Below, we present the results that address our research questions. For each question, we present a table with the mean, median and mode values of students’ learning outcomes related to human teacher’s cognitive feedback (Control Group) and Affective Pedagogical Tutor (Experimental Group). Moreover, in the same table we show the results of t-test to analyze more in deep the differences between GC and EG.

### 5.1 Differences in learning outcomes according to *CF* (RQ1)

In Table 2 (a), we can observe that means in both groups are higher than 3, which seems to indicate that students were satisfied by the *CF* received by both the teacher and the animated agent (APT). Nevertheless, the mode measure provided more information about which *CF* was more significant in each group (GC and EG). In this sense, we can observe that 3.1 was more important to GC and 3.2, 3.3, 3.4, 3.6 and 3.10 were more important in EG.

### 5.2 Differences in learning outcomes according to AF (RQ2)

In Table 2 (b), we can observe that means in both groups are higher than 3, which seems indicate that students were satisfied by the AF received by both the teacher and the animated agent (APT). Nevertheless, the mode measure provided more information about which AF was more significant in each group (GC and EG). In this sense, we can observe that only, 3.11 and 3.16 were more significant for EG and the others were equal significant for both groups.

The results of the t-test presented in Table 2 (t-test ≤0.05) showed significant differences between CG and EG in items 3.12, 3.13, 3.17 and 3.19. Thus, certain types of APT AF increased significantly EG students’ learning outcomes compared to human teacher’s feedback.

All others, which have t-test>0.05, did not show significant differences in both groups (highlighted in bold and marked with an “*”). In this sense, our study should carefully reconsider these items and continue to work for improving APT AF design for supporting students’ learning outcomes.

**Figure 2:**
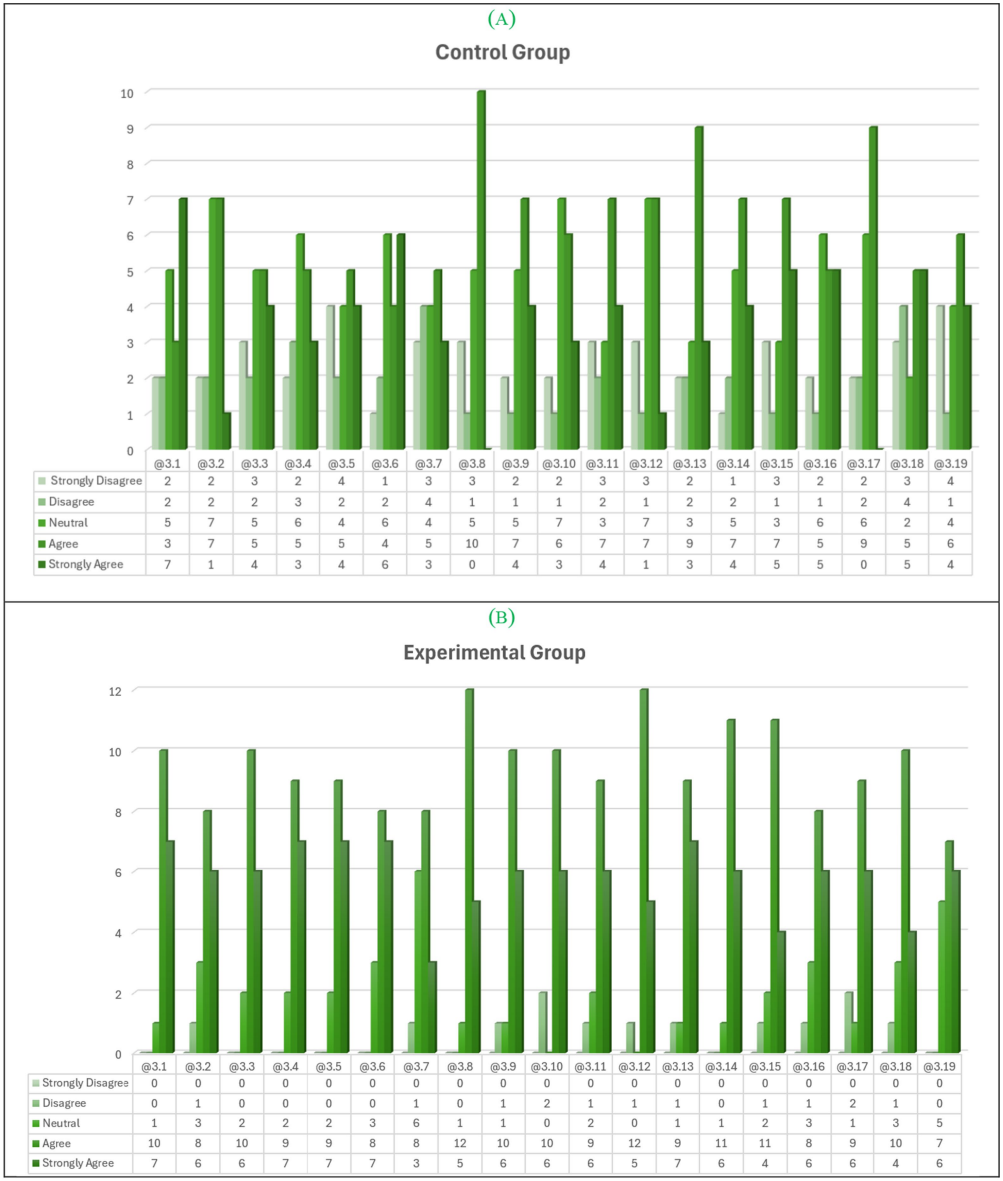
The distribution of responses according to the Likert scale used can be presented in a bar chart, with one bar for each response category to **(A)** CG and **(B)** EG. Axis *Y* shows results obtained for each measure using a 5-point Likert-type scale, ranging from 1 (Strongly Disagree) to 5 (Strongly Agree). Axis *X* shows all items about cognitive and affective feedback.

In addition, we present the distribution of responses according to Likert scale used in GC and EG, respectively, as shown in Figures 2A,B.

## 6 Discussion

The study investigates whether the APT CaAF improves student learning outcomes more effectively than human teacher feedback. The first research objective (RQ1) examined the impact of APT *CF.* The findings show that students who interacted with the animated agent perceived its *CF* as significantly more effective compared to those who received feedback from the teacher. The only exception was feedback related to working in small groups (3.6), where no significant difference was observed.

Unlike previous studies that primarily focused on performance metrics like scores and learning gains (Martha and Santoso, 2019), this study emphasizes students’ perceptions of their learning outcomes. Research indicates that the effectiveness of pedagogical agents depends on specific conditions and features (Schroeder et al., 2017). Challenges in emotion detection mechanisms can limit the agent’s effectiveness (Scholten et al., 2017), but the study’s system aims to offer reliable emotional awareness, enhancing feedback quality. Lin et al. (2020) also highlighted that elaborate feedback leads to higher learning scores, supporting the design choice for APT’s detailed feedback, which proved effective except for group work facilitation.

The learning situation in this study, involved students in a long-term “Web Design” activity. Both the duration and the specificity of the activity acted as a crucial factor that influenced the feedback types which were specifically designed and adapted to this context. This is in line with previous research work, such as Dinçer and Doganay (2017), who have emphasized the importance of customizing agents to fit specific learning environments.

The second objective (RQ2) focused on APT’s AF. Results identified four types of AF (3.12, 3.13, 3.17, and 3.19) that significantly enhanced learning outcomes compared to the teacher’s feedback. These types included guidance for group communication, task completion, addressing difficulties, and maintaining interest. This aligns with previous studies that demonstrate the effectiveness of concise, supportive feedback in reducing negative behaviors and maintaining engagement (Cabestrero et al., 2018).

Several of the AF types used in this study were consistent with empathetic or task-based strategies identified in other research (D’Mello et al., 2011). Text-based AF also proved valuable, taking forms such as prompts, hints, and motivational messages, which positively influenced learning outcomes (Grawemeyer et al., 2017).

## 7 Conclusion

Our findings point out that the APT has an important effect on learning situations which depends on students’ collaborative activities. Although the learning activities were similar in both groups, students that interacted with the APT perceive that their learning is significantly more enhanced compared to learning reported by the students who interacted with the teacher.

First, the majority of *CF* was perceived by the students who interacted with the animated agent as significantly more effective for their learning outcomes compared to students who interacted with the teacher. Second, though students seem satisfied by the AF received by both the teacher and the animated agent (APT), four (out of nine) AF types that students received from the animated agent were perceived as significantly more conducive to their learning outcomes compared to AF received by the teacher. Finally, both CaAF is necessary to act together so that to enhance significantly students’ learning outcomes.

Many agent-based studies have been laboratory-based and the participants were often college students, usually from a university subject pool (Cabestrero et al., 2018). Unlike other studies, our study constitutes an *in-situ* that has integrated specific CaAF strategies into an APT design, aiming at enhancing their learning outcomes.

The results of our experiments on APT effectiveness are drawn from the users’ perceptions, by means of questionnaires. However, a recent review on CPAs reveals a set of CPA design recommendations to promote their use in different learning situations (Pérez-Marín, 2021) that include instructional methods embedded in the agent, new interaction modalities and domains (which may change the type of agent used), as well as Human-Computer Interaction guidelines besides of real-time user signals can be also captured by sensors. The analysis of such data can be fed into the APT endowing it with adaptive and social behavior according to users’ needs and task requirements. Such information can also be used to cross-check students’ learning outcomes that have been provided by the questionnaire.

To further enhance the generalizability and impact of the findings, future studies should expand the testing of APT across diverse academic subjects and learning environments. This would allow a more comprehensive evaluation of how cognitive and affective feedback (CaAF) strategies perform in varied contexts. Moreover, a deeper exploration of the reasons behind the differing effectiveness of specific affective feedback types is recommended. Including case examples and connecting findings to established theories in educational psychology could provide practical insights. Finally, introducing a longitudinal component to assess the durability of the observed improvements in learning outcomes over time would significantly strengthen the study’s contributions. This approach would not only validate the long-term benefits of APT but also highlight its potential for sustained educational enhancement.

## Data Availability

Requests to access the datasets should be directed to martaarg@uoc.edu.
